# Emergence of Lumpy Skin Disease Virus Infection in Yaks, Cattle-Yaks, and Cattle on the Qinghai–Xizang Plateau of China

**DOI:** 10.1155/2024/2383886

**Published:** 2024-07-01

**Authors:** Yuqing Song, Ou Zuo, Gelin Zhang, Jianwu Hu, Zhancheng Tian, Guiquan Guan, Jianxun Luo, Hong Yin, Youjun Shang, Junzheng Du

**Affiliations:** ^1^ State Key Laboratory for Animal Disease Control and Prevention College of Veterinary Medicine, Lanzhou University Lanzhou Veterinary Research Institute Chinese Academy of Agricultural Sciences, Lanzhou 730000, China; ^2^ Gansu Province Research Center for Basic Disciplines of Pathogen Biology, Lanzhou, Gansu 730046, China; ^3^ Animal Epidemic Disease Prevention and Control Center of Agricultural and Rural Bureau of Ali Prefecture, Gaer County, Xizang Autonomous Region 859499, China; ^4^ College of Veterinary Medicine Huazhong Agricultural University, Wuhan 430070, China; ^5^ Jiangsu Co-innovation Center for Prevention and Control of Important Animal Infectious Diseases and Zoonoses Yangzhou University, Yangzhou 225009, China

## Abstract

Lumpy skin disease (LSD) is a viral disease caused by lumpy skin disease virus (LSDV), which mainly infects cattle and can cause huge economic losses. In May 2023, yaks, cattle-yaks, and cattle in Tibet (Xizang), China, developed fever, skin nodules, and severe discharges and were suspected to be cases of LSD. Samples from these animals were analyzed using molecular biology and serological methods. The *RPO30*, *P32*, and *GPCR* genes were amplified by PCR and sequenced, and the whole genome of the virus was determined using viral metagenomics technology. Sequencing results showed that it was indeed an LSDV infection, and enzyme-linked immunosorbent assay results confirmed the presence of LSDV antibodies. The whole genome phylogenetic tree shows that LSDV/CHINA/Tibet/2023 is different from the previous epidemic strains in China, but clusters with India 2022 strain. This is the first report of LSD in yaks, cattle-yaks, and cattle on the highest altitude plateau in the world.

## 1. Introduction

Lumpy skin disease (LSD) is a viral disease that affects a wide variety of ruminants and is caused by lumpy skin disease virus (LSDV). LSDV is a member of the genus *Capripoxvirus*, family Poxviridae and is transmitted by a variety of arthropods, such as hard ticks, stable flies, and mosquitoes, so LSDV outbreaks are related to season and the distribution of arthropods [1, 2]. However, the latest research found that direct or indirect contact transmission is possible [3, 4]. LSD causes clinical signs such as fever, skin and mucosal nodules, and lymphadenopathy in infected animals, which can lead to a sharp decline in milk production, death, and permanent damage to leather [5]. Therefore, LSD can cause huge economic losses, and epidemics are notifiable to the World Organization for Animal Health [6].

Over the past 100 years, LSD has spread from Africa to countries in the Middle East, Eastern Europe, and Asia [7]. LSD was first documented in Zambia in 1929 and was endemic in Africa until it reached Israel in 1989 [8]. LSD was rediscovered in cattle on the Israel–Syria border in 2012 and then spread across the Middle East to Turkey, Iraq, and Iran. It has also spread to Europe: Greece in August 2015, and subsequently Bulgaria, the Republic of Macedonia, Serbia, Kosovo, and Albania [9]. In August 2019, the first outbreak of LSD was described in Xinjiang, China, and other outbreaks of LSD occurred in southeastern China in 2020 [10]. The China GD01/2020 strain (MW355944.1) shows evidence of unique recombination events involving the genetic backbone of the parental vaccine strain LW-1959/vaccine (AF409138.1) and LSDV/KSGPO-240/Kenya/1958 (KS-1; MN072619.1) [11, 12]. Soon, Southeast Asian countries such as Vietnam and Thailand also reported LSD outbreaks [13, 14, 15]. The migration of cattle, trade transportation, and the movements of people contribute to the long-distance transmission of LSDV. The LSD outbreak in Israel is thought to be the result of a viral vector carried by the wind [16]. According to the analysis, cattle trafficking and the influx of 2 million refugees from Syria may have been responsible for the outbreak of LSD in Turkey in 2013 [17].

Recombination of LSDV under natural conditions has been reported. The 1957 South African Neethling LSDV strain and its derived LAV vaccine strain, and the field isolates obtained from Africa, the Middle East, and continental Europe form the two original clusters [18, 19]. Since 2017, eight distinct recombinant strains have been identified [11, 12, 19, 20, 21]. The vaccine-like strain LSDV/RUSSIA/Saratov/2017 (MH646674.1) is the first evidence of genetic exchange between LSDV and vaccine strains found in the field. The strain contains the skeleton of a live attenuated vaccine scattered with wild-type viral DNA fragments [12]. A novel LSDV is not only genetically distinct from the identified LSDV strains, but may also exhibit novel phenotypic features. For example, a recombinant LSDV successfully overwintered in a cold climate and demonstrated more aggressive growth in primary cells and cattle than the classical field isolate [3, 22, 23].

The China 2019 strain spread widely in 14 provinces of China but did not spread in Tibet (Xizang) [24, 25]. Herein, we describe the first outbreak of LSDV in Xizang, China, and cases of LSDV infection in yaks, cattle-yaks, and cattle in the Qinghai–Xizang Plateau. Whole-genome sequencing demonstrated that LSDV/CHINA/Tibet/2023 (Xizang 2023 strain; OR797612.1) was most similar to OQ606832.1 and OQ588787.1, which caused the outbreaks in India in 2022, most likely indicating cross border transmission at high altitudes. This deepens our understanding of the ability of LSDV to spread. With the spread of LSDV, different clades of the LSDV, Xizang 2023 strain and the China 2019 strain, emerged in China, which will increase the risk of viral recombination and difficulties in LSD prevention and control.

## 2. Materials and Methods

### 2.1. Sample Collection and DNA Extraction

Samples were collected and immediately immersed in 1 ml PBS. DNA was extracted from broken skin nodules using a Viral Genomic DNA Extraction Kit (TIANGen, Beijing, China), and DNA was extracted from blood samples using a Blood Genomic DNA Extraction Kit (TIANGen).

### 2.2. PCR and ELISA

As previously published [26], primers targeting the *P32*, *RPO30*, and *GPCR* genes were synthesized (Sangon, Shanghai, China) and used for the detection of sequences in extracted DNA via polymerase chain reaction (PCR). To perform antibody testing, an LSDV competition enzyme-linked immunosorbent assay (ELISA) antibody detection kit (Lanzhou Institute of Biological Products Co., Ltd., China) was employed, following the manufacturer's instructions.

### 2.3. Library Construction and Sequencing

The viral genome was extracted from a 200-*μ*g tissue sample (skin nodules) homogenate. For DNA quantification, a Nanodrop 2000 spectrophotometer (Thermo Fisher Scientific, Waltham, MA, USA) was used to detect the quality values of the viral DNA (A260/280 and A260/230) and later a Qubit 3.0 Fluorometer (Life Technologies corporation, Gaithersburg, MD, USA) with the dsDNA HS (High Sensitivity) Assay Kit (Invitrogen, Carlsbad, CA, USA) was employed, according to the manufacturers' instructions, to obtain a more accurate quantification.

Viral DNA was fragmented with NEBNext® dsDNA Fragmentase® (New England Biolabs, Beverly, MA, USA). DNA fragments were used as input to the NEB “NEBNext® Ultra™ II DNA Library Prep Kit for Illumina®” protocol. An Agilent 2100 Bioanalyzer (Agilent Technologies, Santa Clara, CA, USA), with a HS DNA Kit (Agilent Technologies) was used to quantify the library. The library followed the Illumina protocol “Prepare DNA libraries for sequencing on the NovaSeq 6000”. Paired-end 150 nt reads were generated.

### 2.4. NGS Read Processing and Sequence Assembly

Read quality trimming was performed using the Skewer with an additional trimming filter for unreliable sequences after application of a user-specified quality score [27]. Host read subtraction by read mapping was performed with the bwasw program (version 0.7.9a-r786) against ribosomal RNAs (16, 18, 23, 28, 5S, and internal transcribed spacers rRNA were retrieved from the following ftp site), bacterial genome sequences, and the latest host organism genome sequences [28]. In the full reads, the host- and bacteria-related results are removed, and the remaining data are then spliced for the target virus. The *de novo* assembly followed the A5-miseq pipeline [29]. The final scaffolds were subjected to bwasw read mapping and a mega blast homology search against the NCBI Nucleotide database (https://www.ncbi.nlm.nih.gov/nuccore) [30].

### 2.5. Phylogenetic Analysis

A total of 88 LSDV sequences larger than 139 kbp were searched for and downloaded from the NCBI Nucleotide database as of September 27, 2023 (Table S1). The MAFFT online version (https://mafft.cbrc.jp/alignment/software/) was used for sequence alignment and phylogenetic analysis with the default settings for sequence alignment parameters, 1,000 bootstrap iterations, and the neighbor-joining method [31, 32]. The EMBL-EBI online tool (https://www.ebi.ac.uk/) for pairwise sequence alignment was used to calculate the sequence similarity of Xizang 2023 strain to OM984485.1 and OQ588787.1 [33]. ON400507.1, AF409137.1, MH893760.2, OM984485.1, and OQ588787.1 were obtained from Clade 1.1, Clade 2.1, Clade 2.2.2, Clade 3.1, and Clade 2.2.1, respectively. The single nucleotide polymorphisms (SNPs) were assigned as distinct from the Xizang 2023 strain, and their corresponding genomic positions were annotated using Excel.

### 2.6. Analysis of *GPCR*, *P32*, and *PRO30* Genes

For the analysis of the genes, *PRO30* genes for LSDV were downloaded from the NCBI Nucleotide database (Table S2). Specifically for the 88 LSDV sequences mentioned above, the MAFFT online version [31, 32] was used to extract these *PRO30* genes. Subsequently, all *PRO30* genes were combined for sequence alignment and phylogenetic analysis. Similarly, the sequence data for the *P32* and *GPCR* genes of LSDV were also downloaded and analyzed.

## 3. Result

### 3.1. Clinical Signs

**Figure 1:**
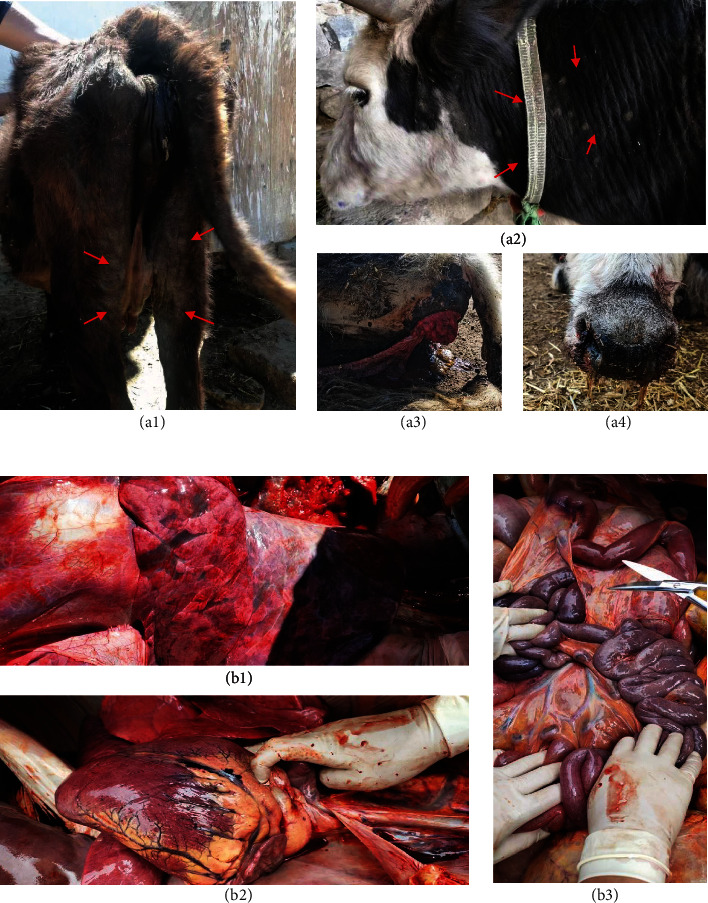
Clinical signs and anatomical diagrams of LSDV-infected cattle. (a) Nodules of different sizes appear on the skin of the limbs (a1) and neck (a2), severe diarrhea causes rectal prolapse (a3), and there is profuse nasal discharge (a4). Nodules are indicated with red arrows. (b) Secondary infection causes severe infection of the lungs (b1) and myocardial infarction (b2), with mesenteric lymphadenopathy (b3).

The first case of the disease was detected in Pulan town, Pulan County, Ali Prefecture, Xizang, China in May 2023. It quickly spread throughout the town within 2 weeks. Yaks, cattle-yaks, and cattle raised in the town became sick, with an overall incidence rate of around 2% despite emergency vaccination, strict disinfection, and control measures. Sick cattle generally experienced elevated body temperature, sometimes reaching as high as 42°C, which lasted for ~5–6 days. The skin of affected cattle developed round bumps, measuring 20–30 mm in diameter or larger, with a distinct boundary. These bumps are not painful to the touch (Figure 1(a)). Initially, the bumps appear on the head, neck, chest, perineum, udder, and extremities. In the later stages of infection, cattle might experience excessive discharge from the eyes and nose, increased oral discharge, and even blindness. The disease also led to myocardial infarction and changes in lung tissue (Figure 1(b)).

### 3.2. PCR and Serological Tests

A total of 30 blood samples and 30 serum samples were collected from 30 affected animals (11 yaks, 14 cattle-yaks, and 5 cattle). Eleven skin nodule samples (from 6 yaks, 3 cattle-yaks, and 2 cattle) were also collected from the 30 animals that were the sources of blood and serum samples. Genomic DNA was extracted from skin nodules and blood, and the *PRO30*, *P32*, and *GPCR* genes were amplified by PCR. Nucleic acid electrophoresis showed that eight of 11 skin nodules were positive and four of 30 blood samples were positive (Table 1). The PCR product was sent for testing, and comparison of the sequencing results showed that the virus was LSDV. The PCR sequencing results were consistent with the whole-genome sequencing results, which indicated that the outbreak was caused by the same strain of LSDV. According to the ELISA results, 27 of the 30 sera tested were positive for antibodies (Table 1).

### 3.3. Genomic Analysis

We sequenced one whole genome of LSDV from one infected cattle-yak using viral metagenomics technology. Illumina sequencing generated around 93 million reads. After quality control, around 91 million clean reads were obtained. After subtraction of ribosomal RNAs and host reads, 247,175 reads remained; of which 27,027 reads were related to viruses. The complete genome of LSDV was obtained. The average depth and median depth of the coverage were 48.3 and 48, respectively. The resulting genome sequence was 150,470 bp in length and was aligned with all of the available LSDV genome sequences for phylogenetic analysis.

**Figure 2:**
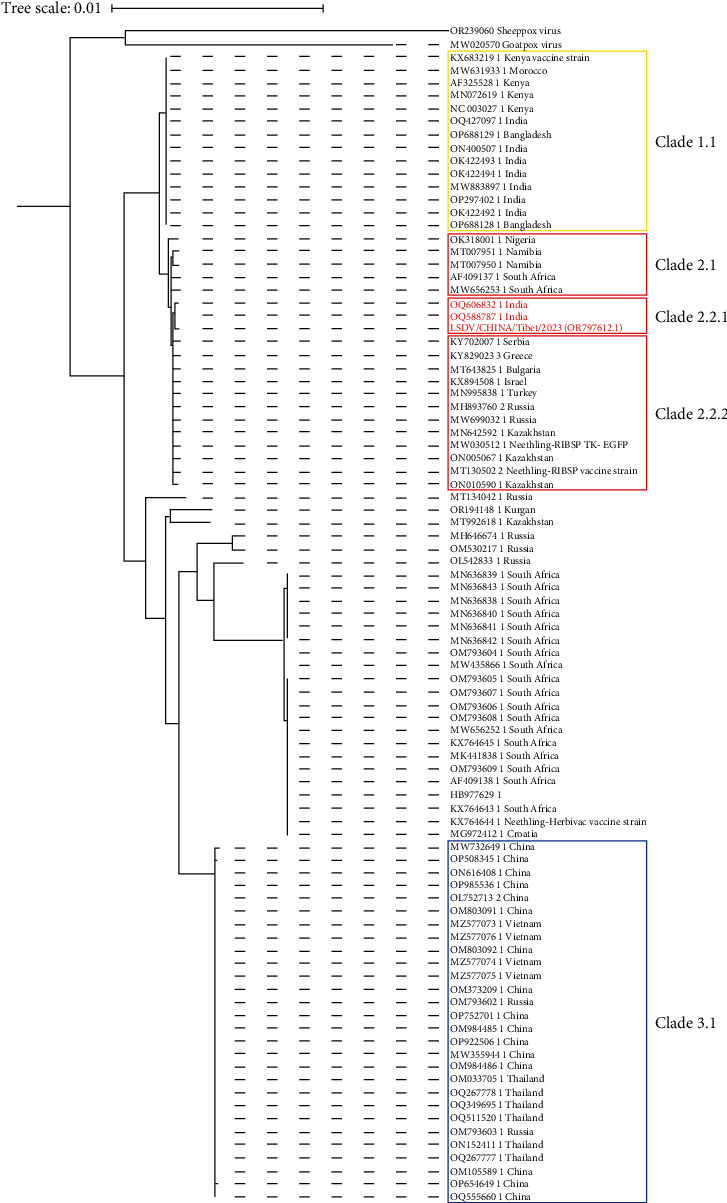
LSDV genome phylogenetic tree. Clade 2 consists of Clade 2.1 from South Africa, Namibia, and Nigeria, Clade 2.2.1 from Xizang and India 2022, and Clade 2.2.2 from Europe and the Middle East. The India 2019 strain belongs to Clade 1.1. The endemic strains from Southeast Asian countries and previous strains from China clustered into Clade 3.1. This suggests that the Xizang 2023 strain is a new clade of LSDV that has emerged in China.

**Figure 3:**
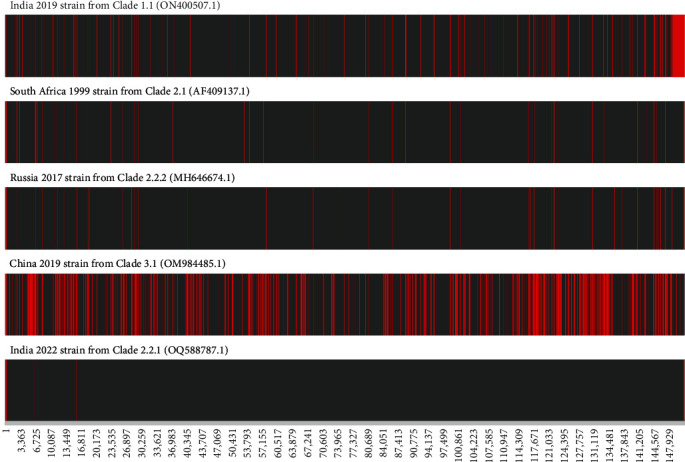
Graphical representation of SNPs in red across the genome of the Xizang 2023 strain. The SNPs identified within the genomes of the India 2019 strain from Clade 1.1 (ON400507.1), South Africa 1999 strain from Clade 2.1 (AF409137.1), Russia 2017 strain from Clade 2.2.2 (MH646674.1), China 2019 strain from Clade 3.1 (OM984485.1), and India 2022 strain from Clade 2.2.1 (OQ588787.1) are presented.

Genome phylogenetic analysis of LSDV showed that Xizang 2023 strain (OR797612.1) is in the same clade (Clade 2.2.1) as India 2022 strain (OQ606832.1 and OQ588787.1), with a similarity of up to 100% (Figure 2). The similarity with the LSDV/CHINA/XJ/2019 strain (OM984485.1) in China was 99.0%. By detection of SNPs, 1924, 383, 373, 1576, and 157 SNPs were identified between the Xizang 2023 strain and the India 2019 strain from Clade 1.1 (ON400507.1), the South Africa 1999 strain from Clade 2.1 (AF409137.1), Russia 2017 strain from Clade 2.2.2 (MH646674.1), China 2019 strain from Clade 3.1 (OM984485.1), and the India 2022 strain from Clade 2.2.1 (OQ588787.1), respectively. The SNPs are indicated at corresponding locations in the genome (Figure 3). It is evident that the Xizang 2023 strain is different from the previous epidemic strains in China.

### 3.4. Phylogenetic Analysis of *GPCR*, *P32*, and *PRO30* Genes

**Figure 4:**
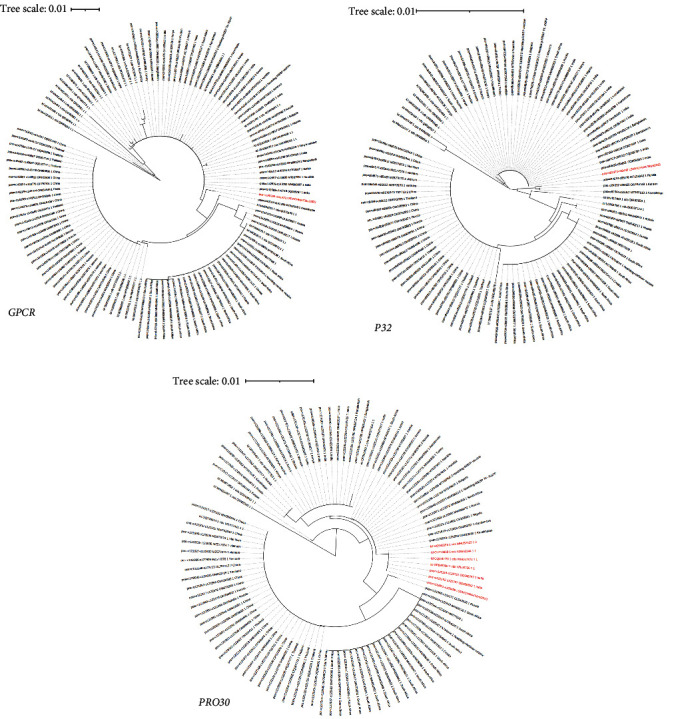
Phylogenetic analysis of the *GPCR*, *P32*, and *PRO30* genes. (a, b) Evolutionary analysis of *GPCR* and *P32* showed that these genes could not distinguish Xizang 2023 strain and the India 2022 strain from other strains. (c) The evolutionary analysis of *PRO30* showed that *PRO30* clustered with the Xizang 2023 strain and India 2022 strain in a single clade.

We downloaded sequences of 98 *PRO30*, 114 *GPCR*, and 98 *P32* genes and plotted their phylogenetic trees (Figure 4). *GPCR* and *P32* gene sequences showed that Clade 1.1, Clade 2.1, Clade 2.2.1, and Clade 2.2.2 were all the same clade. They cannot be distinguished. *PRO30* successfully brought together the Xizang 2023 strain and the India 2022 strain, notably including a sequence from Sudan in 2006. This suggests that *PRO30* may be used as a basis to distinguish Clade 2.2.1 from other strains. The inefficient grouping of some genes and lack of more information on the 2006 Sudan sequence strongly reflected the importance of whole-genome sequencing.

### 3.5. Global Distribution of Different LSDV Clades

**Figure 5:**
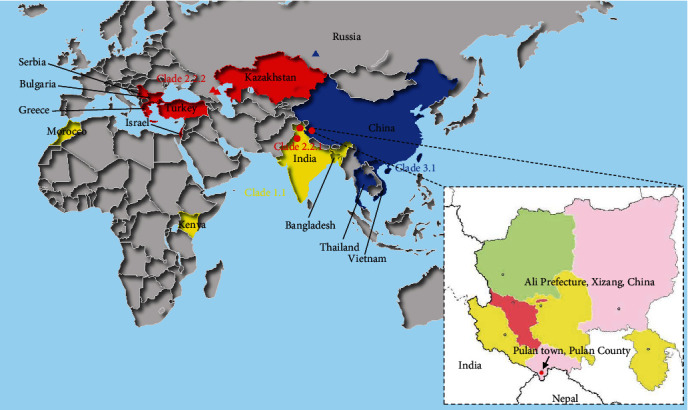
Distribution of different clades of LSDV. The distributions of Clade 1.1, Clade 2.2, and Clade 3.1 are, respectively, represented by areas in yellow, red, and blue (Note: The Russian strains of Clade 2.2.2 and Clade 3.1 are located far away, and they are represented by red and blue triangles.). Clade 2.2.1 is represented by red dots. Diffusion ranges of different clades increase and intersect. Local magnification was performed in Ali Prefecture, Xizang, China (inset). Colored areas represent Ali Prefecture, Xizang, China, and the red dot represents Pulan town where the Xizang 2023 strain emerged.

On the map, we indicate the countries or regions in which the genomes used for sequence analysis were collected (Figure 5). The sequences of KSGP vaccine strain and the Kingdom of Morocco, Kenya, India 2019, and Bangladesh strains are included in Clade 1.1, Xizang 2023 strain and India 2022 strain in Clade 2.2.1 and strains from Serbia, Russia, Greece, Turkey, Israel, and Kazakhstan in Clade 2.2.2 are included in Clade 2.2. Because of the discovery of the India 2022 strain and the Xizang 2023 strain, we were able to find that Clade 1.1 and Clade 2.2.1, Clade 3.1 and Clade 2.2.1 have been present in the same country, which increases the possibility of recombination among different LSDV strains.

## 4. Discussion

In May 2023, we detected the first outbreak of LSD on the Qinghai–Xizang plateau in China, where local yaks, cattle-yaks, and cattle developed typical symptoms. At high altitudes, under harsh natural conditions, and low population density, LSDV still exhibited a rapid propagation rate. This may be related to the warmer temperatures, larger numbers of arthropods, and increased tourism activity at that time.

Xizang 2023 strain (OR797612.1) is highly similar to the India strain isolated in 2022. In 2019, there was an outbreak of LSD in India, and the circulating strains were all ancestral LSDV wild-type strains from the Kenyan lineage [34]. However, in 2022, a unique lineage of LSDV emerged in India. In mid 2022, the mortality rate in the Indian outbreak reached 15%, which was higher than in 2019 [35]. Recently it was reported that an LSDV strain (OP893960) from an Indian gazelle was responsible for the death of the gazelle [36]. *PRO30* brings together Xizang 2023 strain, the India 2022 strain, and this strain (OP893960), suggesting that they may be the same epidemic strain.

The first outbreak of LSD in Xinjiang, China, also occurred in 2019, but genome phylogenetic analysis showed that the India 2019 strain was different from the China 2019 strain [37]. The Xizang 2023 strain also was distantly related to LSDV prevalent in China. The India 2019 strain was placed in Clade 1.1, the Xizang 2023 strain and the India 2022 strain in Clade 2.2.1, and the previous epidemic strains from China in Clade 3.1. The results of the SNP analysis also reflected the phylogenetic relationship between the Xizang 2023 strain and representative strains in other clades. Nepal, which borders Xizang, China, also had an outbreak of LSD in 2020, but no LSDV whole-genome sequences from Nepal were reported, and only sequences of some genes from LSDV strains in 2020 [38]. Analysis of the *PRO30* genes showed that the Xizang strain, and Nepalese strains did not cluster (Figure 4). This indicates that the 2020 LSDV strain in Nepal and Xizang 2023 strain are not the same. Kazakhstan also borders China. Genomic analysis showed that Kazakhstan's LSDV clustered in Clade 2.2.2, which was also different from Xizang 2023 strain. In the phylogenetic analysis, LSDV genomes from Asian countries such as India, Turkey, Israel, Russia, Kazakhstan, Bangladesh, China, Vietnam, and Thailand were used. The Xizang 2023 strain from Clade 2.2.1 was the first to have been found outside of India.

Following the first documented outbreak of LSD in 2019, the emergency use of heterologous live attenuated GTPV (Uttarkashi strain) vaccine was banned in India and the use of other live attenuated LSD vaccines was also banned [34]. It is not clear why Clade 2.2.1 appeared in India. Our analysis shows that different clades of strains coexist and the risks of LSDV recombination are growing. Regular testing and whole-genome sequencing are necessary for analysis of viral evolution and spread.

In summary, we report a novel strain of LSDV that differed from the previous epidemic strains in China. It occurs at high altitudes on the Qinghai–Xizang Plateau and causes disease in yaks, cattle-yaks, and cattle. We presumed that LSDV had spread across national borders to Xizang, China, but evidence is lacking to determine the specific route of its transmission. This case demonstrated for the first time the ability of LSDV to spread on the Qinghai–Xizang Plateau. LSDV of different clades coexist as LSDV spreads widely, and we emphasize the importance of whole-genome sequencing and regular monitoring.

## Figures and Tables

**Table 1:** Results of PCR and serologic tests.

Type of sample	Method of detection	Positive rate
Skin nodule	PCR	8/11
		5/6 yaks, 2/3 cattle-yaks, and 1/2 cattle

Blood	PCR	4/30
		0/11 yaks, 3/14 cattle-yaks, and 1/5 cattle,

Serum	ELISA	27/30
		10/11 yaks, 13/14 cattle-yaks, and 4/5 cattle

## Data Availability

All sequencing data generated in this study have been deposited at the GenBank of National Center for Biotechnology Information (NCBI) under the accession number OR797612.

## Supplementary Materials

Supplementary Materials Table S1: genome sequence information used for LSDV genome phylogenetic tree. Table S2: *GPCR*, *P32*, and *PRO30* genes information used for phylogenetic analysis.
