# Supplementary material for: Emergence of Lumpy Skin Disease Virus Infection in Yaks, Cattle-Yaks, and Cattle on the Qinghai–Xizang Plateau of China
Source: Transbound Emerg Dis. 2024 Jul 1;2024:2383886. doi: 10.1155/2024/2383886 (PMC12016979; doi:10.1155/2024/2383886)
Supplement: Supplementary Materials — Table S1: genome sequence information used for LSDV genome phylogenetic tree. Table S2: GPCR, P32, and PRO30 genes information used for phylogenetic analysis. [file 2383886.f1.docx]

**Table S1.** Genome sequence information used for LSDV genome phylogenetic tree

| **GenBank Accession** | **Gene Length** | **Collection Country** |
| --- | --- | --- |
| OP297402.1 | 150969 | India |
| OM373209.1 | 150873 | China |
| OL752713.2 | 150822 | Kinmen Island, Taiwan, China |
| OQ349695.1 | 150812 | Thailand |
| OQ511520.1 | 150812 | Thailand |
| AF409137.1 | 150793 | Warmbaths, South Africa |
| OP752701.1 | 150776 | Fujian, China |
| OP688129.1 | 150775 | Bangladesh |
| AF325528.1 | 150773 | Kenya |
| NC_003027.1 | 150773 | Kenya |
| OP688128.1 | 150773 | Bangladesh |
| OQ606832.1 | 150763 | India |
| MH893760.2 | 150751 | Russia |
| OP922506.1 | 150726 | China |
| MN995838.1 | 150721 | Turkey |
| MT643825 | 150719 | Yambol region, Bulgaria |
| OM105589.1 | 150697 | Xinjiang, China |
| OM033705.1 | 150689 | Thailand |
| OQ267777.1 | 150689 | Thailand |
| OQ267778.1 | 150689 | Thailand |
| MW435866.1 | 150682 | South Africa |
| KY829023.3 | 150681 | Greece |
| OK318001.1 | 150681 | Nigeria |
| MZ577073.1 | 150665 | Northern Vietnam, Vietnam |
| OM803092.1 | 150665 | Guangxi, China |
| MZ577076.1 | 150664 | Northern Vietnam, Vietnam |
| KX683219.1 | 150663 | Kenya |
| KY702007.1 | 150661 | Serbia |
| MW883897.1 | 150655 | India |
| OP985536.1 | 150644 | Guangdong, China |
| OM984485.1 | 150641 | Xinjiang, China |
| OQ588787.1 | 150611 | Kapran, Anantnag, Kashmir- J&K, India |
| OM803091.1 | 150610 | Guangdong, China |
| MH646674.1 | 150606 | Russia |
| MW355944.1 | 150606 | China |
| ON152411.1 | 150602 | Prachuap Khiri Khan, Thailand |
| MZ577075.1 | 150600 | Vietnam |
| MZ577074.1 | 150599 | Vietnam |
| OM984486.1 | 150592 | Fujian, China |
| MT007950.1 | 150586 | Namibia |
| KX894508.1 | 150562 | Israel |
| OP508345.1 | 150532 | Xinjiang, China |
| KX764644.1 | 150529 | Neethling-Herbivac vaccine |
| MT007951.1 | 150523 | Namibia |
| OM793608.1 | 150522 | South Africa |
| OM793604.1 | 150516 | South Africa |
| MW732649.1 | 150513 | Hong Kong, China |
| AF409138.1 | 150509 | South Africa |
| HB977629.1 | 150509 | / |
| KX764645.1 | 150508 | South Africa |
| OM793607.1 | 150508 | South Africa |
| OM793609.1 | 150508 | South Africa |
| MK441838.1 | 150507 | South Africa |
| OM793605.1 | 150507 | South Africa |
| MN642592.1 | 150485 | Atyrau region, Kazakhstan |
| KX764643.1 | 150480 | South Africa |
| MG972412.1 | 150448 | Croatia |
| ON400507.1 | 150445 | India |
| MT134042.1 | 150436 | Udmurtiya, Russia |
| MN636839.1 | 150398 | South Africa |
| MN636838.1 | 150396 | South Africa |
| MN636840.1 | 150396 | South Africa |
| MN636841.1 | 150396 | South Africa |
| MN636842.1 | 150396 | South Africa |
| MN636843.1 | 150396 | South Africa |
| OM793606.1 | 150370 | South Africa |
| MW699032.1 | 150344 | Dagestan, Russia |
| OL542833.1 | 150338 | Tyumen, Russia |
| MW656253.1 | 150335 | South Africa |
| MN072619.1 | 150329 | Kenya |
| OM530217.1 | 150309 | Saratov, Russia |
| MW656252.1 | 150300 | Haden/RSA/1954, South Africa |
| OP654649.1 | 150236 | China |
| OM793602.1 | 150222 | Toms, Russia |
| OM793603.1 | 150209 | Khabarovsk, Russia |
| ON005067.1 | 148628 | Kazakhstan |
| ON616408.1 | 148453 | China |
| OK422493.1 | 147681 | India |
| OQ427097.1 | 147548 | West_Bengal, India |
| OK422492.1 | 146171 | Ranchi-1, India |
| MT130502.2 | 146159 | Kazakhstan, Neethling-RIBSP vaccine |
| MW631933.1 | 146090 | Morocco |
| MT992618.1 | 145865 | Kazakhstan |
| ON010590.1 | 145838 | Kazakhstan |
| MW030512.1 | 139366 | Neethling-RIBSP(TK-)EGFP |
| OK422494.1 | 139051 | Ranchi-1, India |
| OR194148.1 | 150270 | Kurgan |
| OQ555660.1 | 151411 | China |

**Table S2.** *GPCR*, *P32 and PRO30* genes information used for phylogenetic analysis

| ***GPCR* of GenBank Accession** | ***P32* of GenBank Accession** | ***PRO30* of GenBank Accession** |
| --- | --- | --- |
| KP663706.1 | MN422450.1 | MN422457.1 |
| KP663706.1 | MN422448.1 | MN871852.1 |
| KP663691.1 | MN422447.1 | OP893960.1 |
| FJ869365.1 | MN422451.1 | OQ030223.1 |
| LC573970.1 | MW815879.1 | GU119938.1 |
| KP071937.1 | MN422449.1 | OQ943598.1 |
| KP071936.1 | OQ943604.1 | OQ746914.1 |
| MW452648.1 | LC663765.1 | OP903457.1 |
| MN508357.1 | ON637137.1 | OP903458.1 |
| MK302071.1 |  |  |
| FJ869376.1 |  |  |
| OR137811.1 |  |  |
| OR137810.1 |  |  |
| MZ666003.1 |  |  |
| MZ666002.1 |  |  |
| OM250060.1 |  |  |
| OR209678.1 |  |  |
| OQ355030.1 |  |  |
| MN271737.1 |  |  |

Note: This table has added genes other than those extracted from the genome in Table S1.
